# Cdk5r1 Overexpression Induces Primary *β*-Cell Proliferation

**DOI:** 10.1155/2016/6375804

**Published:** 2015-12-14

**Authors:** Carrie Draney, Amanda E. Hobson, Samuel G. Grover, Benjamin O. Jack, Jeffery S. Tessem

**Affiliations:** Nutrition, Dietetics and Food Science Department, College of Life Sciences, Brigham Young University, Provo, UT 84602, USA

## Abstract

Decreased *β*-cell mass is a hallmark of type 1 and type 2 diabetes. Islet transplantation as a method of diabetes therapy is hampered by the paucity of transplant ready islets. Understanding the pathways controlling islet proliferation may be used to increase functional *β*-cell mass through transplantation or by enhanced growth of endogenous *β*-cells. We have shown that the transcription factor Nkx6.1 induces *β*-cell proliferation by upregulating the orphan nuclear hormone receptors Nr4a1 and Nr4a3. Using expression analysis to define Nkx6.1-independent mechanisms by which Nr4a1 and Nr4a3 induce *β*-cell proliferation, we demonstrated that cyclin-dependent kinase 5 regulatory subunit 1 (Cdk5r1) is upregulated by Nr4a1 and Nr4a3 but not by Nkx6.1. Overexpression of Cdk5r1 is sufficient to induce primary rat *β*-cell proliferation while maintaining glucose stimulated insulin secretion. Overexpression of Cdk5r1 in *β*-cells confers protection against apoptosis induced by etoposide and thapsigargin, but not camptothecin. The Cdk5 kinase complex inhibitor roscovitine blocks islet proliferation, suggesting that Cdk5r1 mediated *β*-cell proliferation is a kinase dependent event. Overexpression of Cdk5r1 results in pRb phosphorylation, which is inhibited by roscovitine treatment. These data demonstrate that activation of the Cdk5 kinase complex is sufficient to induce *β*-cell proliferation while maintaining glucose stimulated insulin secretion.

## 1. Introduction

The incidence of both major types of diabetes is increasing at an alarming rate [1]. Type 1 diabetes is characterized by autoimmune destruction of the pancreatic *β*-cells. Type 2 diabetes, while being initially characterized by decreased insulin sensitivity of peripheral tissues, ultimately results in the loss of *β*-cell mass. Various approaches have been studied to increase functional *β*-cell mass. Islet transplantation has the potential to rescue the *β*-cell deficiency observed in both forms of diabetes. A major obstacle to greater use of islet transplantation is the lack of transplant ready *β*-cells.

Various studies have demonstrated that most *β*-cell proliferation occurs prior to adolescence [2–6]. Interestingly, *β*-cell proliferation has been observed in obese and pregnant human populations [7–9]. These data suggest that while *β*-cell proliferation is tightly controlled the pathway responsible for proliferation is nonetheless intact and given the proper stimuli can be engaged to induce pancreatic islet *β*-cell expansion. Various groups have sought to delineate the molecular pathways responsible for inducing proliferation of adult primary *β*-cells as a potential therapeutic intervention for diabetes. Recent studies have shown that the *β*-cell transcription factor Nkx6.1 induces *β*-cell proliferation by upregulating expression of the orphan nuclear receptors Nr4a1 and Nr4a3 [10, 11]. These data begin to delineate a molecular pathway capable of inducing *β*-cell proliferation that may be leveraged as a therapeutic for enhancing functional *β*-cell mass in diabetic patients.

Cdk5 is a member of the eukaryotic cyclin-dependent kinase superfamily [12]. Binding to the activator component Cdk5r1 activates the Cdk5 kinase complex [13]. The Cdk5/Cdk5r1 complex is highly expressed in neurons and is required for proper central nervous system development and neuronal migration [14]. Cdk5 kinase activity has been shown to regulate synaptic vesicle exocytosis [15]. Furthermore, deregulation of the Cdk5 kinase has been associated with progression of Alzheimer's disease, breast cancer, and prostate cancer [16–18].

Recent studies have shown that Cdk5 and Cdk5r1 are expressed in pancreatic *β*-cells [19]. Studies have illustrated that activated Cdk5 promotes insulin exocytosis and that Cdk5 protects against *β*-cell apoptosis [20–23]. Here we report that Cdk5r1 is upregulated by the orphan nuclear receptors Nr4a1 and Nr4a3 in primary rat islets. We demonstrate that overexpression of Cdk5r1, and not Cdk5, is sufficient to induce *β*-cell proliferation in the 832/13 INS-1 *β*-cell line and primary rat islets while maintaining insulin content and glucose stimulated insulin secretion. Furthermore, we show that Cdk5r1 mediated proliferation is dependent on the Cdk5 complex kinase activity. Finally, we demonstrate that Cdk5r1 mediated proliferation correlates with increased phosphorylation of pRb, which results in activation of *β*-cell cell cycle machinery through release of E2F transcription factors. We propose that the Cdk5r1 mediated *β*-cell proliferation pathway may be harnessed to increase *β*-cell numbers for ultimate islet transplantation therapy.

## 2. Materials and Methods

### 2.1. Animal Husbandry and Islet Isolation

Wistar rat breeding pairs were purchased from Harlan and maintained on standard chow diet (Teklad 7001; Harlan). Pups were weaned at 21 days, at which point female rats were euthanized. Male rats were allowed to feed ad libitum and were maintained on a 12-hour light-dark cycle. Rats were age- and weight-matched for all islet experiments. Pancreatic islets were isolated from 5-week-old male rats as previously described [24–26]. All animal studies were approved and performed in accordance with Brigham Young University's IACUC guidelines.

### 2.2. Islet Culture

Primary rat islets were cultured in RPMI 1640 and supplemented with 10% FBS, 1% Fungizone antimycotic (Life Technologies), and 1% HEPES. Islet medium was changed every 24 hours. For experiments using the Cdk5 inhibitor roscovitine, islets were cultured for 48 hours with 10 *μ*mol/L roscovitine (Santa Cruz) or DMSO (vehicle, Sigma) [27]. For all islet experiments using adenoviral transduction, viral treatments occurred within four hours of completing the islet isolation procedure as previously published [10, 11, 26, 28–32]. Islets were transduced with adenovirus for 18 hours, after which media and adenoviral vectors were removed. All measurements on primary rat islets (proliferation, survival, and insulin secretion) occurred 96 hours after islet isolation.

### 2.3. Adenoviral Cloning and Preparation

Recombinant Cdk5r1 and Cdk5 expressing adenovirus constructs were generated and purified as previously described [33]. Adenoviruses expressing Nkx6.1, Nr4a1, Nr4a3, and GFP have been described elsewhere [10, 34–39]. All recombinant viruses were shown to be replication incompetent by being E1a deficient, using an RT-PCR screen, as previously described [40]. For studies involving adenovirus mediated gene manipulation, pools of 200 islets were transduced with ~2 × 10^7^ IFU/mL adenovirus (moi~100–200) for 18 hours, as previously described [10, 11, 29, 32, 41, 42]. Media were changed after 18 hours and islets were cultured for up to 96 hours. Assays were completed at 48, 72, or 96 hours after harvest depending on the experiment.

### 2.4.  832/13 INS-1 Tissue Culture and Apoptosis Assays

The 832/13 INS-1 cells were a kind gift of Dr. Christopher Newgard, Duke University Medical Center [43]. Cells were cultured as previously described. For apoptosis assays, 832/13 cells were plated at a concentration of 2 × 10^5^ cells/mL in 24-well plates at 1 mL per well. Following 24 hours of incubation, the cells were transduced with ~2 × 10^7^ IFU/mL adenovirus (moi~100–200) of AdCMV-GFP, AdCMV-Cdk5, or AdCMV-Cdk5r1 or left untransduced. Following 48 hours of culture, the cells were treated with etoposide (0.9 *μ*M), camptothecin (2.0 *μ*M), thapsigargin (0.32 mM), or DMSO for 18 hours. Cell number was determined by cell counts. Cells were trypsinized, resuspended in PBS, and counted using a hemocytometer. Cell viability was determined by comparing the percentage of cells remaining after drug treatment to the percentage of cells after DMSO treatment. Total caspase-3 and cleaved caspase-3 were measured by western blot analysis of cells transduced with AdCMV-GFP or AdCMV-Cdk5r1 treated with DMSO (no drug), camptothecin, etoposide, or thapsigargin. Ratio of cleaved caspase-3 versus total caspase-3 was measured and quantified.

### 2.5. siRNA Knockdown of Cdk5

832/13 cells were transfected using Lipofectamine RNAiMAX (Life Technologies) with siRNA duplexes against rat Cdk5, 5′-GUUCAGCCCUCCGGGAGAUTT-3′ [44], or a previously described scrambled control, 5′-GAGACCCTATCCGTGATTA-3′ [32, 34]. Following an overnight incubation, cells were transduced with AdCMV-GFP or AdCMV-Cdk5r1 or left untreated. RNA was harvested to measure extent of Cdk5 knockdown by RT-PCR, and proliferation was determined by cell counts.

### 2.6. [^3^H]-Thymidine Incorporation

DNA synthesis rates were measured as previously described [10, 34]. [Methyl-^3^H]-thymidine was added at a final concentration of 1 *μ*Ci/mL to groups of ~200 islets for the final 48 hours of culture. Groups of 20 islets were picked in triplicate and washed twice in RPMI-1640 with unlabeled thymidine and twice in PBS and then the DNA was precipitated with 500 *μ*L cold 10% trichloroacetic acid and solubilized with addition of 80 *μ*L of 0.3 N NaOH. [^3^H]-thymidine incorporation was measured by liquid scintillation counting and normalized to total cellular protein.

### 2.7. Glucose Stimulated Insulin Secretion

Insulin secretion was measured in triplicate in groups of 20 islets per condition as previously described [10] in secretion assay buffer (SAB) containing 2.5 mM glucose for 1 hour at 37°C (basal) followed by incubation in SAB containing 16.7 mM glucose for 1 hour (stimulatory). Insulin was measured in SAB using the Coat-A-Count kit (Siemens). Islets were lysed in RIPA buffer and total protein was determined by BCA (Pierce), and insulin content was measured as described [10].

### 2.8. Immunoblot Analysis

Clarified cell lysates were run on 4–12% NuPAGE gels (Invitrogen) and transferred to polyvinylidene fluoride (PVDF) membranes. Membranes were probed with diluted antibodies raised against Nkx6.1 (Iowa Developmental Hybridoma Bank, F55A10), GFP (Abcam, ab13970), *γ*-tubulin (Sigma, T5326), Cdk5r1 (Cell Signaling, 2680), Cdk5 (Cell Signaling, 2506), caspase-3 (Cell Signaling, 9665), pRb (Cell Signaling, 9313), or phosphorylated pRb (Cell Signaling, 9309). Sheep anti-mouse (1 : 10,000) and goat anti-rabbit (1 : 10,000) antibodies (GE Healthcare) coupled to horseradish peroxidase were used to detect the primary antibodies. Blots were developed with ECL advance reagent (GE Healthcare). Quantitation of immunoblots was performed using ImageJ.

### 2.9. Quantitative RT-PCR

RNA was harvested using the RNEasy microkit (Qiagen) and cDNA was synthesized in an iScript reaction (BioRad). Real-time PCRs were performed using the Life Technologies One Step Plus Sequence Detection System and Software (Life Technologies). TaqMan-Based Assay on Demand primers and probes (Life Technologies) were used to detect rat Cdk5, Cdk5r1, and peptidylprolyl isomerase A (PPIA, internal control). All primer sequences are available upon request.

### 2.10. Histology and* In Situ *Immunofluorescence

Islets were cultured with EdU at a concentration of 10 *μ*M, with daily media changes, for 96 hours [11]. Pools of 50 islets were dispersed and plated on poly-D lysine coated coverslips (BD). EdU was detected using AlexaFluor 555 EdU cell proliferation kit (Invitrogen). Cells were counterstained with insulin to define *β*-cells and DAPI to define nuclei. Five sections containing greater than 400 nuclei were evaluated from EdU signal evaluated using ImageJ software for each experimental condition.

### 2.11. Gene Ontology Analysis

Previously published microarray data were queried for genes upregulated by Nr4a1 and Nr4a3 48 hours after adenoviral transduction that were not regulated by Nkx6.1 at 24, 48, or 96 hours after adenoviral transduction time points. Genes were selected by being induced by Nr4a1 and Nr4a3, not by Nkx6.1, with 1.5-fold or greater induction and a *p* value less than 0.05. Gene ontology (GO) analysis was completed using Panther pathway analysis, as described.

### 2.12. Statistical Analysis

Data are presented as the mean ± S.E.M. For statistical determinations, data were analyzed by the paired Student's* t*-test or by one-way ANOVA with Bonferroni post hoc analysis for multiple group comparisons.

## 3. Results

### 3.1. Cdk5r1 Is Induced by Nr4a1 and Nr4a3

We have previously shown that overexpression of the *β*-cell transcription factor Nkx6.1 is sufficient to induce *β*-cell proliferation [10]. Nkx6.1 mediated *β*-cell proliferation is dependent on expression of the Nr4a1 and Nr4a3 orphan nuclear receptors [11]. Using microarray analysis, we have shown that many of the Nr4a1 and Nr4a3 upregulated genes are also upregulated by Nkx6.1 at a later time point. Furthermore, knockout of Nr4a1 or Nr4a3 is sufficient to impede expression of Nkx6.1 upregulated genes and abrogate Nkx6.1 mediated *β*-cell proliferation, demonstrating Nkx6.1 dependence on the Nr4a1 and Nr4a3 transcription factors [11]. We sought to determine if any of the Nr4a1 or Nr4a3 upregulated genes is independent of the Nkx6.1 proliferation pathway. To address this question, we used data mining techniques to compare the previous published microarray data to define genes that are induced by Nr4a1 and Nr4a3 but are not included in the Nkx6.1 mediated *β*-cell proliferation pathway. Using our previously published microarray data we defined genes upregulated by Nr4a1 and Nr4a3 after 48 hours of expression which are not induced by Nkx6.1 at the 24-, 48-, or 96-hour time points. From this analysis, we determined that 114 genes were upregulated by Nr4a1 and Nr4a3 by 1.5-fold or greater which are not regulated by Nkx6.1.

**Figure 1:**
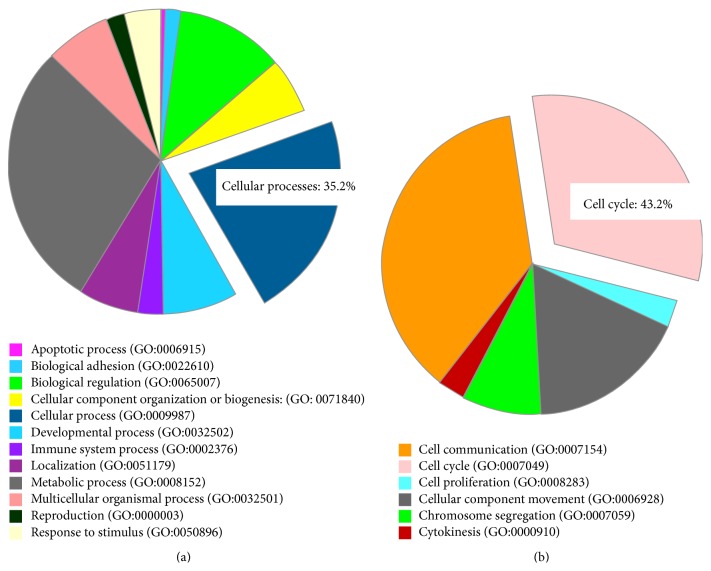
Overexpression of Nr4a1 and Nr4a3 results in upregulation of a subset of cell cycle genes unregulated by Nkx6.1. Previous published microarrays of Nr4a1, Nr4a3, or Nkx6.1 overexpressed in primary rat islets were compared for genes induced by Nr4a1 and Nr4a3 but not induced by Nkx6.1. (a) Using Panther pathway analysis (pantherdb.org), 114 genes were found to be upregulated by Nr4a1 and Nr4a3 but not by Nkx6.1, of which 35.2% were found to be involved in the GO category cellular processes. (b) Within the GO category cellular processes, 43.2% of the genes (17 genes) were found to be associated with the GO category cell cycle.

Using the Panther pathway analysis software, we determined the most highly represented biological functions from our selected genes, based on GO terms. From this analysis, we determined that the strongest upregulated GO biological functions at 49.5% of our genetic subset (54 genes) were metabolic process (which consists of 3 genes in biosynthetic process, 9 genes in catabolic process, 1 gene in coenzyme metabolic process, 1 gene in generation of precursor metabolites and energy, 12 genes in nitrogen compound metabolic process, 14 genes in phosphate-containing compound metabolic process, 43 genes in primary metabolic process, and 2 genes in sulfur compound metabolic process) and at 35.2% (42 genes) were cellular process (which consists of 13 genes in cell communication, 11 genes in cell cycle, 1 gene in cell proliferation, 6 genes in cellular component movement, 3 genes in chromosome segregation, and 1 gene in cytokinesis) (Figure 1(a)).

As our goal was to determine genes involved in *β*-cell proliferation which are unique to the Nr4a nuclear hormone receptors, we queried the cellular processes category for genes associated with the cell cycle. With 43.2% of the subset of genes, the Cell Cycle GO biological function was the second largest category, just behind cell communication at 54.1% (Figure 1(b)). Within the cell cycle set of genes, 16 genes were associated with various parts of the cell cycle including cytokinesis, centromere formation, and microtubule formation. Among these genes, the Cdk5 ligand Cdk5r1 was found to be upregulated 1.7-fold by Nr4a1 and Nr4a3 but not upregulated by Nkx6.1. The binding partner of Cdk5r1, Cdk5, was unregulated by Nr4a1, Nr4a3, and Nkx6.1 (Table 1). Cdk5r1, due to its key role as a CDK regulator and the activity of the CDK5 complex in diabetes, was chosen as a potential candidate for further studies.

**Figure 2:**
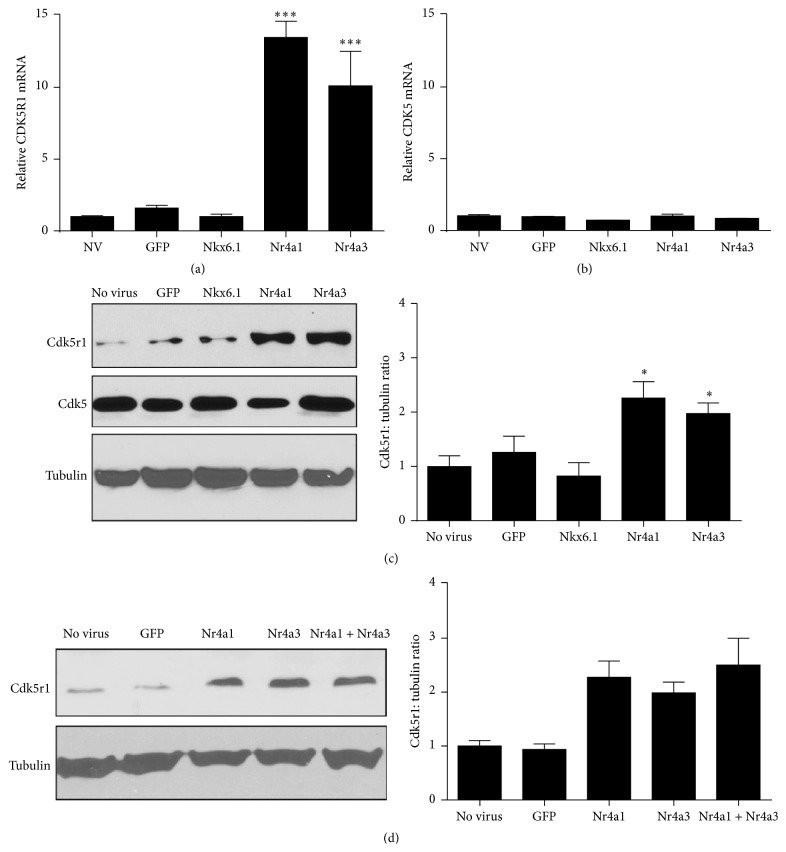
Overexpression of Nr4a1 and Nr4a3 induces Cdk5r1 expression in primary rat islets. Rat islets were treated with AdCMV-GFP, AdCMV-Nkx6.1, AdCMV-Nr4a1, or AdCMV-Nr4a3. (a) Cdk5r1 mRNA levels increase is induced by Nr4a1 and Nr4a3 but not by Nkx6.1, while (b) Cdk5 mRNA levels are unchanged by overexpression of Nkx6.1, Nr4a1, or Nr4a3. Data represent the mean ± SEM of six independent experiments. (c) Representative western blot and quantitation demonstrate that islets transduced with AdCMV-Nr4a1 or AdCMV-Nr4a3 induce upregulation of Cdk5r1 protein levels. Data represent the mean ± SEM of six independent experiments. (d) Representative western blot and quantitation demonstrate that islets transduced with AdCMV-Nr4a1 and AdCMV-Nr4a3 have no additive increase on Cdk5r1 protein levels. Data represent the mean ± SEM of three experiments for western blots. ^*∗*^ *p* ≤ 0.05; ^*∗∗∗*^ *p* ≤ 0.001.* p* value represents the comparison between Nr4a1- or Nr4a3-treated and GFP-treated islets.

To verify the microarray analysis results, we measured Cdk5r1 mRNA from primary rat islets. Islets were transduced with AdCMV-GFP, AdCMV-Nkx6.1, AdCMV-Nr4a1, or AdCMV-Nr4a3 or left untreated. Total RNA was isolated 48 hours after viral transduction. While untreated islets, islets expressing GFP, and islets expressing Nkx6.1 had no increased Cdk5r1 mRNA levels, islets transduced with Nr4a1 or Nr4a3 had 13.5- and 10.1-fold increase, respectively (Figure 2(a)). In contrast, Cdk5 levels do not change when comparing untreated primary rat islets or islets expressing GFP, Nk6.1, Nr4a1, or Nr4a3 (Figure 2(b)). Overexpression of Nr4a1 or Nr4a3 is sufficient to induce increased Cdk5r1 protein levels (Figure 2(c)). Finally, overexpression of Nr4a1 and Nr4a3 in the same islet population demonstrated no synergistic increase in Cdk5r1 protein levels, which is consistent with the highly overlapping transcriptional profiles induced by these transcription factors in primary rat islets (Figure 2(d)). These data demonstrate that Cdk5r1 upregulation is unique to Nr4a1 and Nr4a3 induction and not induced in the Nkx6.1 mediated pathway.

### 3.2. Overexpression of Cdk5r1 Induces Proliferation of Primary Rat Islets

**Figure 3:**
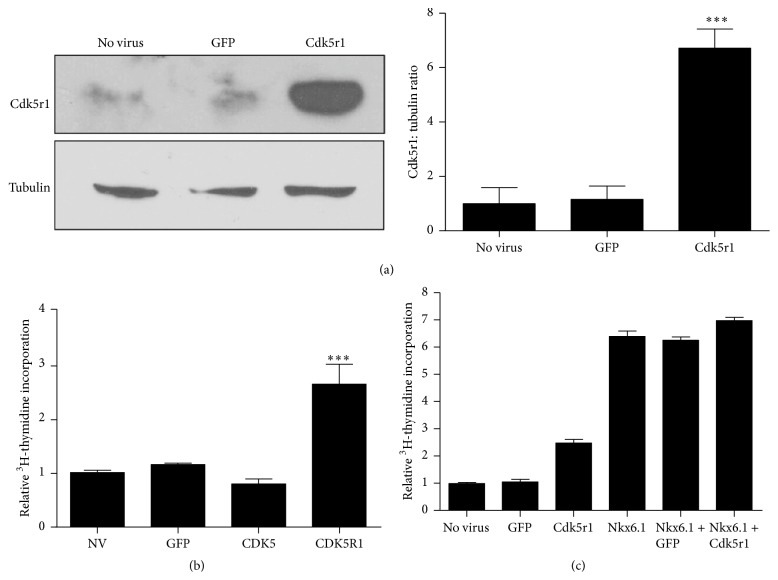
Overexpression of Cdk5r1 is sufficient to induce primary rat islet proliferation. (a) Islets were transduced with AdCMV-GFP or AdCMV-Cdk5r1. Protein was harvested 96 hours after viral transduction. A 6-fold increase was observed in Cdk5r1 protein levels in islets transduced with AdCMV-Cdk5r1, as compared to the observed low endogenous level in primary rat islets. Data represent the mean ± SEM of six independent experiments representing the comparison between untreated islets and islets transduced with AdCMV-Cdk5r1. (b) Incorporation of [^3^H-methyl]-thymidine in rat islets. Rat islets transduced with AdCMV-Cdk5r1 have increased proliferation, while islets treated with AdCMV-GFP or AdCMV-Cdk5 have no induction of proliferation. Data represent the mean ± SEM of four independent experiments representing the comparison between untreated islets and islets transduced with AdCMV-Cdk5r1. (c) Islets were transduced with AdCMV-GFP, Cdk5r1, or Nkx6.1 or were transduced with AdCMV-Nkx6.1 and either GFP or Cdk5r1. Islets were labeled with ^3^H-thymidine 72 hours after viral transduction, followed by measurements of proliferation at 96 hours after viral transduction. Data represent the mean ± SEM of four independent experiments representing the comparison between AdCMV-Nkx6.1 treated islets and islets cotransduced with AdCMV-Nkx6.1 and AdCMV-Cdk5r1. ^*∗*^ *p* ≤ 0.05; ^*∗∗∗*^ *p* ≤ 0.001.

To determine if Cdk5r1 is sufficient to induce islet proliferation, adenoviral vectors expressing Cdk5r1 and Cdk5 were designed. Primary rat islets were cultured without adenoviral transduction or were transduced with AdCMV-GFP, AdCMV-Cdk5, or AdCMV-Cdk5r1 at isolation for 24 hours. Islets were cultured in the presence of ^3^H-thymidine beginning 48 hours after adenoviral transduction and were harvested after 96 hours of culture. As anticipated, transduction with AdCMV-GFP did not enhance islet proliferation. Islets transduced with AdCMV-Cdk5 also failed to induce islet proliferation. Islets transduced with AdCMV-Cdk5r1 demonstrated sixfold induction in Cdk5r1 protein level (Figure 3(a)). This resulted in a 2.5-fold induction in islet proliferation rate (Figure 3(b)). In addition, islets transduced with AdCMV-Cdk5r1 and AdCMV-Nkx6.1 demonstrated an additive effect in terms of proliferation, suggesting that Cdk5r1 overexpression results in the activation of factors that are left unchanged by Nkx6.1 alone, potentially demonstrating that different portions of replication competent pathways are in effect (Figure 3(c)). These data demonstrate that Cdk5r1 is sufficient to induce islet proliferation. Furthermore the inability of Cdk5 overexpression to induce proliferation in primary rat islets suggests that either Cdk5r1 is acting in a Cdk5 independent manner or sufficient Cdk5 protein levels are present in the islet and that addition of Cdk5r1 is necessary to activate the Cdk5-Cdk5r1 proliferation pathway resulting in induction of proliferation.

### 3.3. Overexpression of Cdk5r1 Is Sufficient to Induce *β*-Cell Proliferation

**Figure 4:**
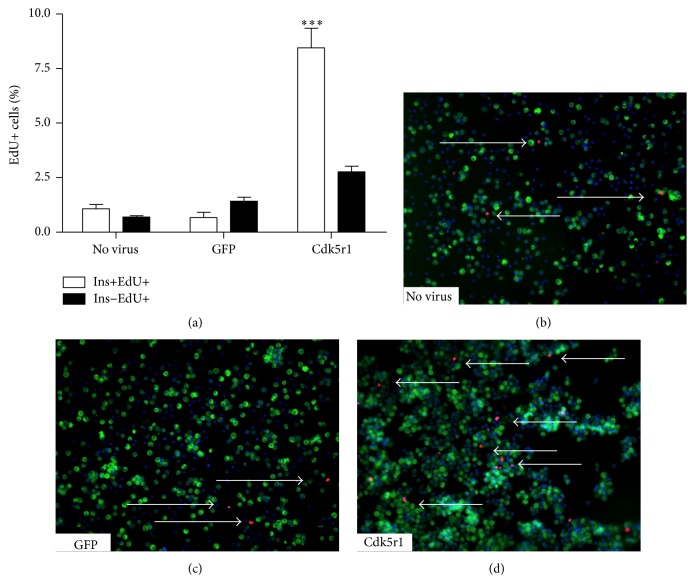
Overexpression of Cdk5r1 is sufficient to induce *β*-cell proliferation. (a) Percentage of BrdU+Insulin+ islet cells cultured with BrdU for 96 hours. (b) Representative image (40x magnification) of dispersed rat islets transduced with AdCMV-GFP labeled with BrdU. (c) Representative image (40x magnification) of dispersed rat islets transduced with AdCMV-Cdk5r1 labeled with BrdU. Arrows indicate BrdU+Insulin+ nuclei. DAPI is blue, insulin is green, and BrdU is red. Data represent the mean ± SEM of four independent experiments. ^*∗∗*^ *p* ≤ 0.01.* p* value represents the comparison between Cdk5r1- and GFP-treated islets.

Our data demonstrate that Cdk5r1 overexpression drives proliferation in primary rat islets. To determine if *β*-cells specifically replicate in response to Cdk5r1 overexpression, we cultured primary rat islets transduced with AdCMV-GFP or AdCMV-Cdk5r1 with the thymidine analog EdU. Islets were cultured in the presence of EdU for 96 hours, after which islets were dispersed and the percentage of Insulin+Edu+ and Insulin−EdU+ cells were calculated. Our findings demonstrate that untransduced islets and islets transduced with AdCMV-GFP had less than 1% of cells positive for EdU in the insulin positive and insulin negative populations (Figures 4(a)–4(c)). In contrast, islets transduced with AdCMV-Cdk5r1 had 8.57% Insulin+EdU+ cells and 2.80% Insulin−EdU+ cells (Figures 4(a) and 4(c)). These data demonstrate that adenoviral mediated expression of Cdk5r1 specifically induces *β*-cell proliferation. Furthermore, given that the majority of proliferation is observed in *β*-cells and not *α*-cells suggests that *β*-cells may express a higher level of Cdk5 than the other islet endocrine cells.

### 3.4. Overexpression of Cdk5r1 Protects 832/13 INS-1 *β*-Cells from Apoptosis

**Figure 5:**
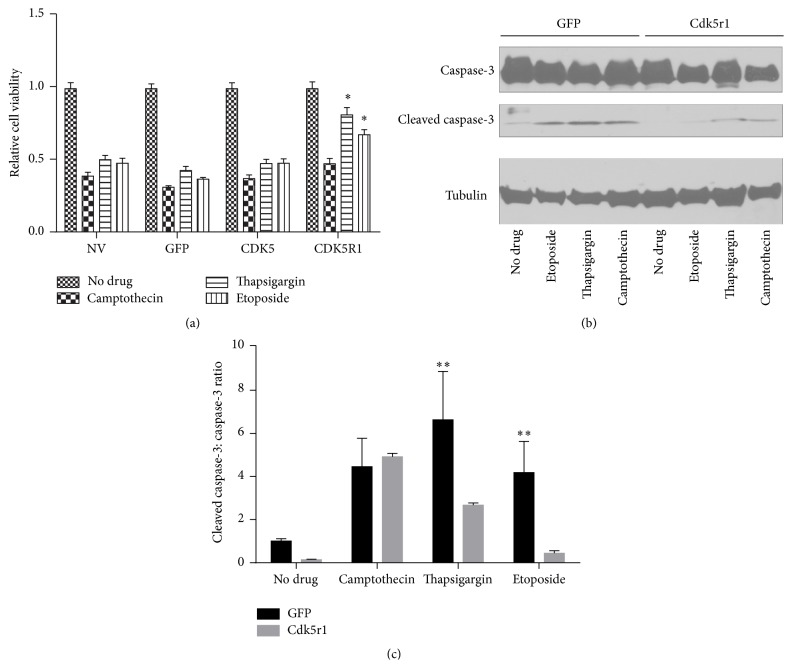
Cdk5r1 overexpression protects INS-1 *β*-cells from apoptosis. (a) 832/13 INS-1 cells were transduced with AdCMV-GFP, AdCMV-Cdk5, or AdCMV-Cdk5r1. Twenty-four hours after adenoviral transduction, cells were cultured for 18 hours in the presence of camptothecin, thapsigargin, etoposide, or the vehicle control. Cell counts were completed 18 hours after drug treatment. Cell viability was determined by comparing the untreated population to the treated population. Cells overexpressing Cdk5r1 presented greater viability when treated with thapsigargin or etoposide than control cells. Data represent the mean ± SEM of nine independent experiments.* p* value represents the comparison between Cdk5r1- and GFP-treated 832/13 cells. Cells were transduced with AdCMV-GFP or AdCMV-Cdk5r1 and subsequently treated with camptothecin, thapsigargin, or etoposide. Western blotting for total caspase-3 or cleaved caspase-3 was queried to determine activation of apoptosis pathway. Representative western blot (b) and quantitation (c). Data represent the mean ± SEM of four independent experiments. ^*∗*^ *p* ≤ 0.01; ^*∗∗*^ *p* ≤ 0.01.

Knockdown of Cdk5 and Cdk5r1 has been shown to result in enhanced caspase-3 cleavage and induction of apoptosis in the 832/13 INS-1 cell line [23]. Based on these findings, we hypothesized that overexpression of Cdk5r1 would result in protection against apoptotic stimuli such as etoposide, thapsigargin, and camptothecin. To determine the effect of Cdk5r1 overexpression on these inducers of apoptosis, untreated cells were compared to cells transduced with AdCMV-GFP, AdCMV-Cdk5, or AdCMV-Cdk5r1. Following a 48-hour culture, the cells were exposed to doses of etoposide, thapsigargin, or camptothecin for 18 hours. Cell viability assays demonstrated that Cdk5r1 overexpression protected 832/13 INS-1 cells treated with thapsigargin or etoposide as compared to untreated cells or cells transduced with AdCMV-GFP or AdCMV-Cdk5 (Figure 5(a)). This demonstrates that Cdk5r1 overexpression protects *β*-cells from Ca^2+^ induced ER stress and topoisomerase II inhibitor induced apoptosis. Interestingly, no protection was observed with Cdk5r1 or Cdk5 in cells treated with camptothecin, an inhibitor of topoisomerase I (Figure 5(a)).

In addition to measuring cell viability through cell counts, we also measured total and cleaved caspase-3 levels. Caspase-3 is activated through cleavage during progression of the apoptotic pathway [31]. A decrease in cleaved caspase-3 levels would indicate decreased activation of the apoptotic pathways. We demonstrated that cells treated with AdCMV-GFP had significantly higher levels of cleaved caspase-3 than cells transfected with AdCMV-Cdk5r1 when both cell types were treated with thapsigargin or etoposide. Cells treated with camptothecin showed no decrease in cleaved caspase-3 levels, supporting our cell viability data for Cdk5r1 and this compound. Taken together, these data demonstrate that overexpression of Cdk5r1 can protect *β*-cellsagainst selected apoptotic stimuli and inhibits caspase-3 activation.

### 3.5. Overexpression of Cdk5r1 Maintains Insulin Content and Secretion in Primary Rat Islets

**Figure 6:**
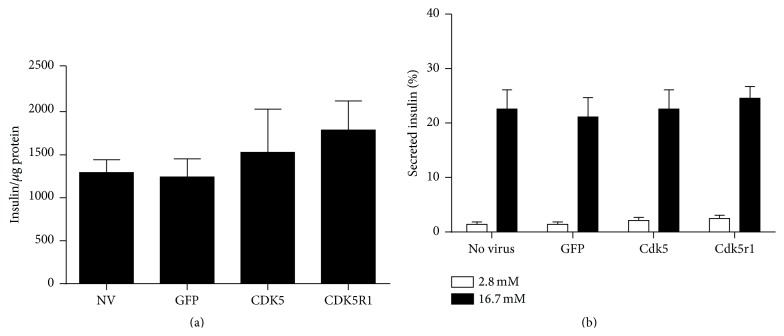
Overexpression of Cdk5r1 maintains glucose stimulated insulin secretion. (a) Total insulin content was measured in primary rat islets at 96 hours after transduction with AdCMV-GFP, AdCMV-Cdk5, or AdCMV-Cdk5r1. No significant difference was observed in total insulin content. (b) Glucose stimulated insulin secretion was measured 96 hours after transduction with AdCMV-GFP, AdCMV-Cdk5, or AdCMV-Cdk5r1 in media containing 2.5 mM glucose and 16.7 mM glucose for 1 hour. No decrease in glucose stimulated insulin rate was observed. Data represent the mean ± SEM of four independent experiments.

Various studies have demonstrated that induction of *β*-cell proliferation is frequently accompanied by decreased insulin secretion [45, 46]. Furthermore, recent studies have demonstrated a connection between the Cdk5 kinase complex and insulin secretion rates [22]. Therefore, we sought to determine the effect of Cdk5r1 overexpression on *β*-cell insulin content and secretion. Islets were transduced overnight with AdCMV-GFP, AdCMV-Cdk5, or AdCMV-Cdk5r1 and subsequently cultured for 96 hours. These populations were compared to untreated islets that were cultured for the same time. Total insulin content of islet populations was measured, and no significant difference was observed between the three treated populations and the untreated controls (Figure 6(a)). Similarly, secreted insulin was measured from adenoviral treated or untreated control islets cultured in the presence of low (2.8 mM) and high (16.7 mM) glucose concentrations. These data also demonstrate that the adenoviral treated islets have similar insulin secretion to the untreated controls (Figure 6(b)). Taken together, these data demonstrate that Cdk5r1 overexpression in primary rat islets, following a 96-hour incubation period, results in maintenance of insulin content and insulin secretion.

### 3.6. Cdk5r1 Mediated *β*-Cell Proliferation Is Dependent on Cdk5 Kinase Activity

**Figure 7:**
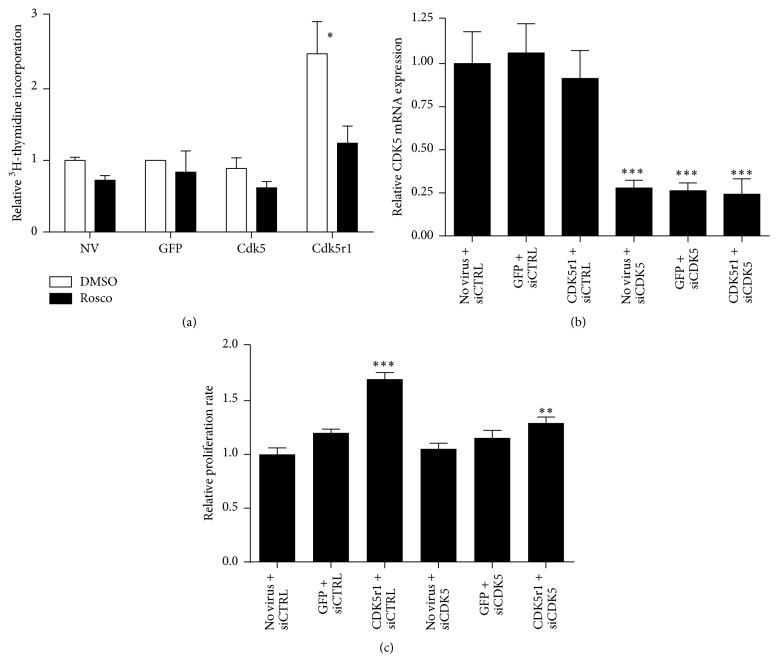
Cdk5r1 mediated *β*-cell proliferation is dependent on the Cdk5 kinase activity. (a) Islets transduced with AdCMV-GFP, AdCMV-Cdk5, or AdCMV-Cdk5r1 were cultured in the presence of ^3^H-thymidine for 48 hours. Islets were treated with vehicle or 10 *μ*M roscovitine for the final 24 hours of the labeling period. Data represent the mean ± SEM of four independent experiments. (b) 832/13 cells were transfected with siCDK5 or siCTRL. Twenty-four hours later cells were transduced with AdCMV-GFP or AdCMV-Cdk5r1. Cdk5 mRNA was measured by RT-PCR, demonstrating a significant decrease in Cdk5 levels with siCDK5 transfection. (c) Proliferation was measured by cell counts, demonstrating that knockdown of Cdk5 causes a significant impairment of Cdk5r1 mediated proliferation. Data represent the mean ± SEM of three independent experiments. (c) ^*∗*^ *p* ≤ 0.05.* p* value represents the comparison between Cdk5r1- and GFP-treated islets.

The primary function of Cdk5r1 is to serve as the binding partner of Cdk5, thus activating the Cdk5: Cdk5r1 kinase activity. We therefore hypothesized that Cdk5r1 mediated proliferation is dependent on Cdk5 kinase activity. To test this hypothesis, islets were transduced with AdCMV-GFP, AdCMV-Cdk5, or AdCMV-Cdk5r1 at isolation and compared to untreated islets. Islets were cultured in the presence of ^3^H-thymidine 48 hours after isolation and were harvested 96 hours after isolation. 72 hours after isolation, the Cdk5 inhibitor roscovitine was added for a 24-hour period [27]. Roscovitine, while being able to inhibit other cdks such as Cdk2 (IC50 of 0.7 *μ*M) and Cdc2 (IC50 of 0.65 *μ*M), has a high affinity for Cdk5 (IC50 of 0.16 *μ*M) [47]. Uninfected AdCMV-GFP and AdCMV-Cdk5 treated islets showed no changes in proliferation rate when treated with roscovitine. Islets transduced with AdCMV-Cdk5r1 had the expected induction in proliferation, which was decreased to control levels when cultured with roscovitine (Figure 7(a)). These data demonstrate that Cdk5r1 mediated proliferation is dependent on the Cdk5: Cdk5r1 kinase activity, and that Cdk5r1 is essential for this kinase to be activated in primary rat *β*-cells.

### 3.7. Knockdown of Cdk5 Inhibits Cdk5r1 Mediated *β*-Cell Proliferation

To determine if knockdown of Cdk5 is sufficient to inhibit the proliferative capacity of Cdk5r1, we used siRNA mediated Cdk5 knockdown in the INS-1 832/13 *β*-cell line. Using a siCDK5 sequence that has previously been shown to be sufficient to knockdown Cdk5 expression in the INS-1 cell line [44], we were able to successfully decrease Cdk5 mRNA expression by 75% (Figure 7(b)). Cells were transfected with siCDK5 or a scrambled siRNA sequence (siCTRL), and 24 hours later the cells were transduced with AdCMV-GFP or AdCMV-Cdk5r1. Cells transduced with AdCMV-Cdk5r1 and transfected with siCTRL had a significant increase in proliferation (Figure 7(c)). Interestingly, cells that were transfected with siCDK5 and transduced with AdCMV-Cdk5r1 had a significant decrease in the proliferation rate. These data support the roscovitine data in demonstrating using a genetic ablation model that Cdk5r1 mediated proliferation is dependent on the Cdk5 kinase activity.

### 3.8. Overexpression of Cdk5r1 in Primary *β*-Cells Results in Increased pRb Phosphorylation

**Figure 8:**
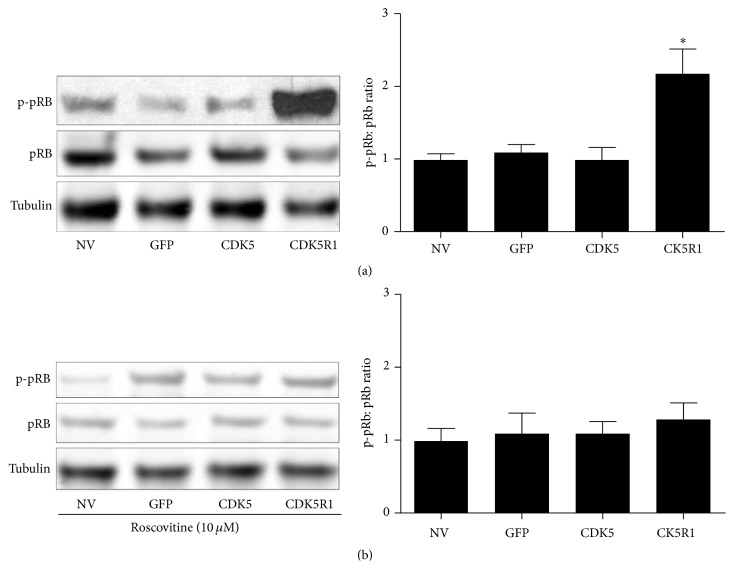
Overexpression of Cdk5r1 results in increased pRb phosphorylation. (a) Untransduced islets were compared to islets transduced with AdCMV-GFP, AdCMV-Cdk5, or AdCMV-Cdk5r1, and levels of phosphorylated pRb were measured. Representative western blot from four independent experiments. (b) Untreated islets and islets transduced with AdCMV-GFP, AdCMV-Cdk5, or AdCMV-Cdk5r1 were treated with 10 *μ*M roscovitine and levels of phosphorylated pRb were measured. Representative western blots and quantitation from three independent experiments. ^*∗*^ *p* ≤ 0.05; ^*∗∗*^ *p* ≤ 0.01; ^*∗∗∗*^ *p* ≤ 0.001.

Cdk5: Cdk5r1 has been shown to phosphorylate pRb, which is sufficient to engage the cell cycle machinery through release of E2F1 and family members in various cell types [48, 49]. We hypothesized that the Cdk5r1 mediated proliferation may be dependent on pRb phosphorylation, which would then engage the cell cycle machinery and permit *β*-cell proliferation. First, we measured the phosphorylated pRb and total pRb levels in untreated islets and islets transduced with AdCMV-GFP, AdCMV-Cdk5, and AdCMV-Cdk5r1. Our data demonstrate that while pRb levels do not significantly change with transduction of any of the adenoviral constructs, the phosphorylated level of pRb significantly increased in islets transduced with AdCMV-Cdk5r1 (Figure 8(a)). Interestingly, culture of islets in the presence of roscovitine significantly decreased the phosphorylation level of pRb in the AdCMV-Cdk5r1 transduced islets (Figure 8(b)). This data demonstrates that Cdk5r1 increases the p-pRb level in a Cdk5 dependent manner. These data suggest that Cdk5: Cdk5r1 mediated phosphorylation of pRb may be essential for Cdk5r1 mediated *β*-cell proliferation.

## 4. Discussion

Control of *β*-cell replication is a highly regulated process, with the majority of *β*-cell replication occurring during embryogenesis and during the neonatal period [2]. Studies have shown, however, that adult *β*-cell expansion does occur during obesity and pregnancy [8, 9, 23]. These data demonstrate that this tightly controlled process is intact in *β*-cells and suggest that defining the molecular components of the *β*-cell proliferation cascade may be used to determine entry points, whereby *β*-cell replication can be induced. These pathways could be leveraged to treat diabetes by inducing *β*-cell replication* ex vivo* for islet transplantation or* in vivo* for expansion of endogenous *β*-cell mass. Therefore, it is imperative to define the molecules involved in known *β*-cell proliferation pathways.

**Figure 9:**
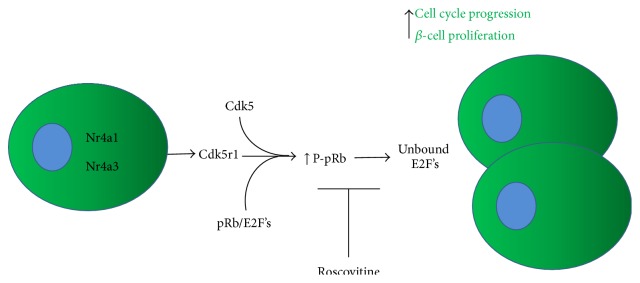
Model of how Cdk5r1 induces *β*-cell proliferation. Overexpression of Nr4a1 or Nr4a3 induces expression of Cdk5r1 in primary rat *β*-cells. Cdk5r1 forms a complex with Cdk5, thus activating the Cdk5 kinase. The Cdk5r1/Cdk5 kinase complex phosphorylates pRb. In the unphosphorylated state pRb binds and sequesters E2F family members, thus inhibiting cell cycle progression. Upon phosphorylation of pRB, E2F family members are released and permitted to induce expression of cell cycle genes that are sufficient to induce proliferation. The Cdk5 kinase inhibitor roscovitine blocks phosphorylation of pRb and inhibits Cdk5r1 induced *β*-cell proliferation.

We have previously shown that the Nkx6.1 mediated *β*-cell proliferation pathway is dependent on the orphan nuclear receptors Nr4a1 and Nr4a3 [10, 11]. Many of the genes upregulated in the Nkx6.1 pathway are directly regulated through the transcriptional activity of Nr4a1 and Nr4a3. Interestingly, upon comparing the gene expression profiles for Nkx6.1 treated islets with Nr4a1 or Nr4a3 treated islets, it is clear that there is a significant subset of genes induced by Nr4a1 and Nr4a3 that are not regulated in the defined Nkx6.1 proliferation pathway. From this subset of genes we have demonstrated that the unique cell cycle activator Cdk5r1 is upregulated by Nr4a1 and Nr4a3 and that Cdk5r1 is sufficient to induce proliferation of primary rat *β*-cells and 832/13 INS-1 cells while maintaining GSIS. Furthermore, we have shown that overexpression of Cdk5r1 in 832/13 INS-1 cells protects against apoptosis induced by thapsigargin and etoposide but not camptothecin. Finally, we have shown that the Cdk5r1 dependent increase in *β*-cells proliferation is dependent on the Cdk5: Cdk5r1 kinase activity. The increase in proliferation corresponds with increased phosphorylation of the cell cycle inhibitor pRb, which results in release and activation of E2F family members. These data demonstrate that expression of Cdk5r1 is sufficient to induce *β*-cells proliferation by releasing a cell cycle braking mechanism (Figure 9).

Expression of Cdk5 and Cdk5r1 was first observed in neuronal cells [14, 50]. Recent studies have clearly demonstrated that both factors are expressed in primary *β*-cells [13, 27]. Since the observation that both Cdk5 and Cdk5r1 are expressed in *β*-cells, much work has been done to address their function in normal and diseased *β*-cells [23, 44, 46]. To date, while other groups have looked at the effect of the Cdk5 complex on glucose stimulated insulin secretion and cellular apoptosis, we are the first to show that Cdk5r1 overexpression and activation of the Cdk5: Cdk5r1 complex result in increased *β*-cell proliferation.

Our data demonstrates that overexpression of Cdk5r1 in primary rat islets results in increased *β*-cell proliferation and that there is no significant increase in proliferation of other non-insulin expressing islet cells. Given that Cdk5r1 activates the Cdk5 kinase complex, these data suggest that *α*-cells and other islet endocrine cells may not express appreciable levels of Cdk5. While no studies have been published to determine the Cdk5 expression level in the non-insulin expressing endocrine cells, our data supports the hypothesis that Cdk5 expression may be sequestered to *β*-cells, thus explaining why overexpression of Cdk5r1 induces proliferation in only the insulin expressing islet population. Furthermore, endogenous levels of Cdk5r1 appear to be very low in *β*-cells (Figure 2(c)). We propose that the low levels of endogenous Cdk5r1 keep the Cdk5 complex in an inactive state. This is typical of cyclin-dependent kinase family, of which Cdk5 is a member.

Several groups have studied the role of the Cdk5 kinase complex on insulin secretion. Within these studies, there are some contradictory findings. Early findings demonstrated that Cdk5 overexpression enhanced insulin secretion from *β*-cells by facilitating vesicle fusion [46]. Latter studies presented data that Cdk5 kinase activity is increased in *β*-cells after culturing with elevated glucose and cytokine levels and that this increase results in decreased insulin gene expression and secretion [22, 44, 51]. Our findings demonstrate that overexpression of Cdk5r1 in primary rat islets maintains glucose stimulated insulin secretion and that total insulin levels are maintained. The differences in our findings may be explained by a number of experimental dissimilarities. Our studies were in primary rat islets that were cultured for 96 hours after adenoviral transduction. Many of the other studies were completed primarily in cell lines or used short-term culture conditions [22, 27, 44, 51]. We did not culture our islets under damaging conditions, such as long-term elevated glucose, or in the presence of cytokines. While our studies were not conducted in a way to determine the effect of endogenous Cdk5r1 in a diseased *β*-cell state, they do clearly demonstrate that Cdk5r1 overexpression is sufficient to induce *β*-cell proliferation while maintaining insulin content and the glucose stimulated insulin secretion response.

In addition to studying the effect of the Cdk5 complex on insulin secretion, other groups have studied the effect on protection from apoptosis. An early study showed in the MIN-6 cell line that overexpression of Cdk5r1 and culture with high glucose resulted in increased apoptosis [52]. A subsequent study using IAPP and *β*-1 integrin signal blockade as induction for *β*-cell apoptosis demonstrated that Cdk5 and Cdk5r1 were necessary for protection against apoptosis [23]. Our data, using Cdk5r1 overexpression, demonstrates that while no protection was observed for apoptosis induced by camptothecin, a significant level of protection was observed in cells overexpressing Cdk5r1 that were treated with etoposide or thapsigargin. While the studies with MIN-6 cells measured Cdk5r1 induced apoptosis [52], our studies indicated no increase in basal death rates. Furthermore, our studies indicated protection against apoptotic stimuli, which was not measured in the previous study. These data demonstrate that Cdk5r1 overexpression is sufficient to confer protection to select apoptotic stimuli in addition to enhancing cellular replication.

Each of the apoptotic inducing compounds used in this study has a unique mechanism by which it induces apoptosis. Thapsigargin blocks the SERCA pumps, thus increasing cytosolic Ca^2+^ levels and inducing ER stress. Camptothecin and etoposide inhibit topoisomerases I and II, respectively. Our data are supported by studies of Cdk5 kinase activity in the literature. Neuroblastoma cells treated with camptothecin had attenuated apoptosis levels when also treated with the Cdk5 inhibitor roscovitine [53]. These data demonstrate that Cdk5 kinase activity is needed for camptothecin-induced apoptosis, which correlates with our findings that Cdk5r1 overexpression fails to confer protection. Studies using etoposide have demonstrated that Cdk5r1 sequestration by C53 results in enhanced apoptosis and C53 deletion resulted in protection to etoposide induced apoptosis [54]. Finally, a recent study demonstrated that PC12 cells treated with the SERCA inhibitor thapsigargin had potentiation of apoptosis when coupled with roscovitine treatment, demonstrating the role of the Cdk5 complex to inhibit Ca^2+^ flux. Our data do not define a mechanism by which Cdk5 kinase activity confers a protective effect against thapsigargin or etoposide induced apoptosis; however a recent study demonstrated that the proapoptotic protein Noxa is phosphorylated by the Cdk5 kinase complex, which results in decreased apoptosis. Future studies will address the mechanism by which Cdk5r1 overexpression protects against these apoptotic stimuli.

Various studies have shown that the activated Cdk5 complex can phosphorylate pRb [48, 55, 56]. This results in release of E2F family members. E2F family members, such as E2F1, E2F2, and E2F3, are critical for engaging the cell cycle by upregulating expression of key genes such as Cyclin E, Cyclin A, and Cyclin B [49]. Studies using neuronal cell lines have shown that Cdk5 mediated phosphorylation of pRb results in reengagement of the cell cycle and induction of neuronal apoptosis [48, 55]. This is in contrast to our findings that pRb phosphorylation is correlated with increased proliferation rates. Previous studies that overexpressed E2F family members in primary *β*-cells have failed to observe enhanced apoptosis rates [57, 58]. These previous findings and our presented data demonstrate that while the default for Cdk5 mediated cell cycle entry in postmitotic neurons may be induction of apoptotic pathways, the same pathway in *β*-cells results in cell cycle reentry and cellular proliferation.

Our data clearly demonstrate that Cdk5r1 has relatively low expression in *β*-cells under normal conditions. Our data demonstrate that overexpression of Nr4a1 or Nr4a3 is sufficient to induce expression of Cdk5r1 and that overexpression of Cdk5r1 is sufficient to induce *β*-cell proliferation. Studies from other groups have demonstrated that Cdk5r1 expression is elevated under high glucose culture conditions [44, 51]. These data are consistent with a proposed role for Nr4a1 and Nr4a3 as cellular nutrient sensors. Expression and activity of the Nr4a orphan nuclear receptors have been shown to increase in various tissues when cultured with elevated glucose, free fatty acid, cholesterol, and amino acid levels [59, 60]. These findings suggest that Cdk5r1 expression may increase during the early stages of type 2 diabetes, presumably through glucose mediated activation of the Nr4a orphan nuclear receptors. During this pathological period, increases in *β*-cell proliferation have been observed [7–9, 23], presumably due to the elevated blood glucose level. Our data may suggest a mechanism by which the initial elevated blood glucose levels increase Nr4a nuclear receptor expression and activity, thus leading to induction of Cdk5r1 expression and the initial proliferation observed during this period of disease progression.

Our findings demonstrate for the first time that overexpression of Cdk5r1 is sufficient to induce *β*-cell proliferation. This is a novel finding that suggests potential uses for* ex vivo* expansion of *β*-cell mass for islet transplantation or* in vivo *induction of endogenous *β*-cell proliferation. Defining the complete cadre of Cdk5 kinase *β*-cell targets will be essential for understanding the activity of this kinase complex for inducing *β*-cell proliferation.

## 5. Conclusions

In conclusion, our findings demonstrate that the orphan nuclear receptors Nr4a1 and Nr4a3 induce expression of the novel cell cycle activator Cdk5r1 independent of the Nkx6.1 signaling pathway. We demonstrate that Cdk5r1 is sufficient to induce *β*-cell proliferation in the 832/13 INS-1 derived cell line and in primary rat islets. We show that Cdk5r1 results in *β*-cell specific proliferation and that this proliferation is dependent on the Cdk5: Cdk5r1 kinase complex activity. Finally, we demonstrate that Cdk5r1 induced *β*-cell proliferation corresponds with increased phosphorylation of the cell cycle inhibitor pRB (Figure 9).

These data demonstrate that expression of Cdk5r1 results in activation of endogenous levels of Cdk5 in *β*-cells resulting in pRB phosphorylation and activation of cell cycle progression. Therefore, our results demonstrate that activation of the novel Cdk5: Cdk5r1 cell cycle kinase may be used as a potential therapeutic entry point for increasing *β*-cell mass* ex vivo* for islet transplantation therapy or expansion of endogenous *β*-cell mass* in vivo *as a treatment for diabetes. This is the first time that overexpression of Cdk5r1 has been shown to be sufficient to induce primary *β*-cell proliferation. Future studies will address how overexpression of Nr4a family members results in activation of Cdk5r1 and other phosphotargets of the Cdk5-Cdk5r1 kinase complex.

## Figures and Tables

**Table 1:** Cell cycle associated genes upregulated by Nr4a1 and Nr4a3 but not Nkx6.1. Genes upregulated by Nr4a1 and Nr4a3 that are not regulated by Nkx6.1 were selected based on changes greater than 1.5-fold and *p* values less than 0.05. Genes were defined by the GO category cell cycle.

Gene name	Fold change	*p* value	Process
*Gas2*	12.27	0.001577852	Cytokinesis
*Tgfa*	2.4	0.000103799	Growth factor
*Rapgef5*	2.31	0.004103808	Ras activator
*Nuf2*	2.24	0.002117132	Centromere
*Chl1*	2.17	0.023016307	G2/M transition
*Top2a*	2.04	0.002104745	DNA replication
*Cks2*	1.92	0.007478331	CDK regulation
*Gins4*	1.87	0.003569736	DNA replication
*Aspm*	1.83	0.004862977	Spindle formation
***Cdk5r1***	**1.78**	**0.00289215**	**CDK regulation**
*Prc1*	1.75	0.005448853	Cytokinesis
*Kif2c*	1.75	0.000350709	Centromere
*Dscc1*	1.73	0.008438809	DNA replication
*Kif15*	1.66	0.008896449	Microtubules
*Ncapd2*	1.65	0.004007301	DNA condensation
*Prim1*	1.58	0.001821116	DNA replication

## References

[1] Weir G. C., Bonner-Weir S. (2004). Five of stages of evolving *β*-cell dysfunction during progression to diabetes. *Diabetes*.

[2] Perl S. Y., Kushner J. A., Buchholz B. A. (2010). Significant human beta-cell turnover is limited to the first three decades of life as determined by in vivo thymidine analog incorporation and radiocarbon dating. *Journal of Clinical Endocrinology and Metabolism*.

[3] Granger A., Kushner J. A. (2009). Cellular origins of *β*-cell regeneration: a legacy view of historical controversies. *Journal of Internal Medicine*.

[4] Rankin M. M., Kushner J. A. (2009). Adaptive beta-cell proliferation is severely restricted with advanced age. *Diabetes*.

[5] Rankin M. M., Kushner J. A. (2010). Aging induces a distinct gene expression program in mouse islets. *Islets*.

[6] Teta M., Long S. Y., Wartschow L. M., Rankin M. M., Kushner J. A. (2005). Very slow turnover of beta-cells in aged adult mice. *Diabetes*.

[7] Butler A. E., Cao-Minh L., R. Galasso (2010). Adaptive changes in pancreatic beta cell fractional area and beta cell turnover in human pregnancy. *Diabetologia*.

[8] Butler A. E., Galasso R., Matveyenko A., Rizza R. A., Dry S., Butler P. C. (2010). Pancreatic duct replication is increased with obesity and type 2 diabetes in humans. *Diabetologia*.

[9] Saisho Y., Butler A. E., Manesso E., Elashoff D., Rizza R. A., Butler P. C. (2013). *β*-Cell mass and turnover in humans: effects of obesity and aging. *Diabetes Care*.

[10] Schisler J. C., Fueger P. T., Babu D. A. (2008). Stimulation of human and rat islet beta-cell proliferation with retention of function by the homeodomain transcription factor Nkx6.1. *Molecular and Cellular Biology*.

[11] Tessem J. S., Moss L. G., Chao L. C. (2014). Nkx6.1 regulates islet beta-cell proliferation via Nr4a1 and Nr4a3 nuclear receptors. *Proceedings of the National Academy of Sciences of the United States of America*.

[12] Malumbres M. (2014). Cyclin-dependent kinases. *Genome Biology*.

[13] Arif A. (2012). Extraneuronal activities and regulatory mechanisms of the atypical cyclin-dependent kinase Cdk5. *Biochemical Pharmacology*.

[14] Kawauchi T. (2014). Cdk5 regulates multiple cellular events in neural development, function and disease. *Development Growth and Differentiation*.

[15] Xin X., Ferraro F., Bäck N., Eipper B. A., Mains R. E. (2004). Cdk5 and Trio modulate endocrine cell exocytosis. *Journal of Cell Science*.

[16] Hsu F.-N., Chen M.-C., Lin K.-C. (2013). Cyclin-dependent kinase 5 modulates STAT3 and androgen receptor activation through phosphorylation of ser727 on STAT3 in prostate cancer cells. *The American Journal of Physiology—Endocrinology and Metabolism*.

[17] Shukla V., Skuntz S., Pant H. C. (2012). Deregulated Cdk5 activity is involved in inducing Alzheimer's disease. *Archives of Medical Research*.

[18] Goodyear S., Sharma M. C. (2007). Roscovitine regulates invasive breast cancer cell (MDA-MB231) proliferation and survival through cell cycle regulatory protein cdk5. *Experimental and Molecular Pathology*.

[19] Ahmed D., Sharma M. (2011). Cyclin-dependent kinase 5/p35/p39: a novel and imminent therapeutic target for diabetes mellitus. *International Journal of Endocrinology*.

[20] Kono T., Ahn G., Moss D. R. (2012). PPAR-*γ* activation restores pancreatic islet SERCA2 levels and prevents *β*-cell dysfunction under conditions of hyperglycemic and cytokine stress. *Molecular Endocrinology*.

[21] Sakamaki J.-I., Fu A., Reeks C. (2014). Role of the SIK2-p35-PJA2 complex in pancreatic *β*-cell functional compensation. *Nature Cell Biology*.

[22] Wei F.-Y., Nagashima K., Ohshima T. (2005). Cdk5-dependent regulation of glucose-stimulated insulin secretion. *Nature Medicine*.

[23] Daval M., Gurlo T., Costes S., Huang C.-J., Butler P. C. (2011). Cyclin-dependent kinase 5 promotes pancreatic beta-cell survival via Fak-Akt signaling pathways. *Diabetes*.

[24] Milburn J. L., Hirose H., Lee Y. H. (1995). Evidence for induction of functional, morphologic, and metabolic abnormalities by increased long chain fatty acids. *Journal of Biological Chemistry*.

[25] Naber S. P., McDonald J. M., Jarett L., McDaniel M. L., Ludvigsen C. W., Lacy P. E. (1980). Preliminary characterization of calcium binding in islet-cell plasma membranes. *Diabetologia*.

[26] Bain J. R., Schisler J. C., Takeuchi K., Newgard C. B., Becker T. C. (2004). An adenovirus vector for efficient RNA interference-mediated suppression of target genes in insulinoma cells and pancreatic islets of langerhans. *Diabetes*.

[27] Kitani K., Oguma S., Nishiki T.-I. (2007). A Cdk5 inhibitor enhances the induction of insulin secretion by exendin-4 both in vitro and in vivo. *Journal of Physiological Sciences*.

[28] Iype T., Francis J., Garmey J. C. (2005). Mechanism of insulin gene regulation by the pancreatic transcription factor Pdx-1: application of pre-mRNA analysis and chromatin immunoprecipitation to assess formation of functional transcriptional complexes. *The Journal of Biological Chemistry*.

[29] Fueger P. T., Schisler J. C., Lu D. (2008). Trefoil factor 3 stimulates human and rodent pancreatic islet *β*-cell replication with retention of function. *Molecular Endocrinology*.

[30] Stephens S. B., Schisler J. C., Hohmeier H. E. (2012). A VGF-derived peptide attenuates development of type 2 diabetes via enhancement of islet beta-cell survival and function. *Cell Metabolism*.

[31] Hayes H. L., Moss L. G., Schisler J. C. (2013). Pdx-1 activates islet *α*- and *β*-cell proliferation via a mechanism regulated by transient receptor potential cation channels 3 and 6 and extracellular signal-regulated kinases 1 and 2. *Molecular and Cellular Biology*.

[32] Hobson A., Draney C., Stratford A. (2015). Aurora Kinase A is critical for the Nkx6.1 mediated *β*-cell proliferation pathway. *Islets*.

[33] Becker T. C., Noel R. J., Coats W. S. (1994). Use of recombinant adenovirus for metabolic engineering of mammalian cells. *Methods in Cell Biology*.

[34] Schisler J. C., Jensen P. B., Taylor D. G. (2005). The Nkx6.1 homeodomain transcription factor suppresses glucagon expression and regulates glucose-stimulated insulin secretion in islet beta cells. *Proceedings of the National Academy of Sciences of the United States of America*.

[35] Chao L. C., Wroblewski K., Zhang Z. (2009). Insulin resistance and altered systemic glucose metabolism in mice lacking Nur77. *Diabetes*.

[36] Chao L. C., Bensinger S. J., Villanueva C. J., Wroblewski K., Tontonoz P. (2008). Inhibition of adipocyte differentiation by Nur77, Nurr1, and Nor1. *Molecular Endocrinology*.

[37] Chao L. C., Zhang Z., Pei L., Saito T., Tontonoz P., Pilch P. F. (2007). Nur77 coordinately regulates expression of genes linked to glucose metabolism in skeletal muscle. *Molecular Endocrinology*.

[38] Pei L., Waki H., Vaitheesvaran B., Wilpitz D. C., Kurland I. J., Tontonoz P. (2006). NR4A orphan nuclear receptors are transcriptional regulators of hepatic glucose metabolism. *Nature Medicine*.

[39] Pei L., Castrillo A., Chen M., Hoffmann A., Tontonoz P. (2005). Induction of NR4A orphan nuclear receptor expression in macrophages in response to inflammatory stimuli. *Journal of Biological Chemistry*.

[40] Lavine J. A., Raess P. W., Davis D. B. (2010). Contamination with E1A-positive wild-type adenovirus accounts for species-specific stimulation of islet cell proliferation by CCK: a cautionary note. *Molecular Endocrinology*.

[41] Tessem J. S., Jensen J. N., Pelli H. (2008). Critical roles for macrophages in islet angiogenesis and maintenance during pancreatic degeneration. *Diabetes*.

[42] Hayes H. L., Moss L. G., Schisler J. C. (2013). Pdx-1 activates islet *α*- and *β*-cell proliferation via a mechanism regulated by transient receptor potential cation channels 3 and 6 and extracellular signal-regulated kinases 1 and 2. *Molecular and Cellular Biology*.

[43] Hohmeier H. E., Mulder H., Chen G., Henkel-Rieger R., Prentki M., Newgard C. B. (2000). Isolation of INS-1-derived cell lines with robust ATP-sensitive K+ channel-dependent and -independent glucose-stimulated insulin secretion. *Diabetes*.

[44] Ubeda M., Rukstalis J. M., Habener J. F. (2006). Inhibition of cyclin-dependent kinase 5 activity protects pancreatic beta cells from glucotoxicity. *The Journal of Biological Chemistry*.

[45] Hohmeier H. E., Newgard C. B. (2005). Islets for all?. *Nature Biotechnology*.

[46] Lilja L., Yang S.-N., Webb D.-L., Juntti-Berggren L., Berggren P.-O., Bark C. (2001). Cyclin-dependent Kinase 5 promotes insulin exocytosis. *The Journal of Biological Chemistry*.

[47] Meijer L., Borgne A., Mulner O. (1997). Biochemical and cellular effects of roscovitine, a potent and selective inhibitor of the cyclin-dependent kinases cdc2, cdk2 and cdk5. *European Journal of Biochemistry*.

[48] Futatsugi A., Utreras E., Rudrabhatla P., Jaffe H., Pant H. C., Kulkarni A. B. (2012). Cyclin-dependent kinase 5 regulates E2F transcription factor through phosphorylation of Rb protein in neurons. *Cell Cycle*.

[49] Li F. X., Zhu J. W., Tessem J. S. (2003). The development of diabetes in E2f1/E2f2 mutant mice reveals important roles for bone marrow-derived cells in preventing islet cell loss. *Proceedings of the National Academy of Sciences of the United States of America*.

[50] Tanaka H., Morimura R., Ohshima T. (2012). Dpysl2 (CRMP2) and Dpysl3 (CRMP4) phosphorylation by Cdk5 and DYRK2 is required for proper positioning of Rohon-Beard neurons and neural crest cells during neurulation in zebrafish. *Developmental Biology*.

[51] Ubeda M., Kemp D. M., Habener J. F. (2004). Glucose-induced expression of the cyclin-dependent protein kinase 5 activator p35 involved in Alzheimer's disease regulates insulin gene transcription in pancreatic *β*-cells. *Endocrinology*.

[52] Zheng Y.-L., Hu Y.-F., Zhang A. (2010). Overexpression of p35 in Min6 pancreatic beta cells induces a stressed neuron-like apoptosis. *Journal of the Neurological Sciences*.

[53] Pizarro J. G., Folch J., Junyent F. (2011). Antiapoptotic effects of roscovitine on camptothecin-induced DNA damage in neuroblastoma cells. *Apoptosis*.

[54] Jiang H., Luo S., Li H. (2005). Cdk5 activator-binding protein C53 regulates apoptosis induced by genotoxic stress via modulating the G2/M DNA damage checkpoint. *The Journal of Biological Chemistry*.

[55] Zhang J., Li H., Yabut O., Haley F., D'Arcangelo G., Herrup K. (2010). Cdk5 suppresses the neuronal cell cycle by disrupting the E2F1-DP1 complex. *The Journal of Neuroscience*.

[56] Lee K.-Y., Helbing C. C., Choi K.-S., Johnston R. N., Wang J. H. (1997). Neuronal Cdc2-like kinase (Nclk) binds and phosphorylates the retinoblastoma protein. *Journal of Biological Chemistry*.

[57] Rady B., Chen Y., Vaca P. (2013). Overexpression of *E2F3* promotes proliferation of functional human *β* cells without induction of apoptosis. *Cell Cycle*.

[58] Grouwels G., Cai Y., Hoebeke I. (2010). Ectopic expression of E2F1 stimulates beta-cell proliferation and function. *Diabetes*.

[59] Close A. F., Rouillard C., Buteau J. (2013). NR4A orphan nuclear receptors in glucose homeostasis: a minireview. *Diabetes and Metabolism*.

[60] Pearen M. A., Muscat G. E. O. (2010). Minireview: nuclear hormone receptor 4A signaling: implications for metabolic disease. *Molecular Endocrinology*.

